# Thickened Retinal Nerve Fiber Layers Associated With High-Altitude Headache

**DOI:** 10.3389/fphys.2022.864222

**Published:** 2022-05-04

**Authors:** Xianhong Yin, Yi Li, Yanyun Ma, Yuan Xie, Kun Wang, Dayan Sun, Xiaoyu Liu, Meng Hao, Meng Liang, Shixuan Zhang, Yuan Guo, Li Jin, Ningli Wang, Jiucun Wang

**Affiliations:** ^1^ Ministry of Education Key Laboratory of Contemporary Anthropology, School of Life Sciences, and Human Phenome Institute, Fudan University, Shanghai, China; ^2^ Institute for Six-Sector Economy, Fudan University, Shanghai, China; ^3^ International Human Phenome Institutes, Shanghai, China; ^4^ Beijing Tongren Eye Center, Beijing Tongren Hospital, Capital Medical University, Beijing, China; ^5^ Research Institute of Data Sciences, Fudan University, Shanghai, China; ^6^ Research Unit of Dissecting the Population Genetics and Developing New Technologies for Treatment and Prevention of Skin Phenotypes and Dermatological Diseases, Chinese Academy of Medical Sciences (2019RU058), Shanghai, China

**Keywords:** headache, high-altitude headache, HAH, retinal nerve fiber layer, RNFL, OCT

## Abstract

**Purpose:** This study aimed to quantify the different quadrants of the optic nerve head (ONH) and macular parameters and their changes during exposure to high altitude, and to assess their correlation with high-altitude headache (HAH).

**Methods:** Spectral-domain optical coherence tomography (OCT) was used to quantify changes in the retinal structure in 109 healthy subjects during acute exposure to high altitude (3,700 m). Self-reported symptoms of HAH and acute mountain sickness AMS were assessed using Lake Louise Score (LLS), alongside measurements of physiological parameters (oxygen saturation [SpO_2_], heart rate [HR], hemoglobin level [Hb], and red blood cell [RBC] count). Measurements were taken before and after exposure to the high-altitude environment. The correlations of these parameters and changes at ONH were examined.

**Results:** With the exposure to high altitude, the incidence of AMS was 44.0% and the frequency of HAH was 67.0% (54.1% mild, 12.9% moderate-severe). As for systemic parameters measured at high altitude, the participants exhibited significantly lower SpO_2_, higher resting HR, higher Hb, and a higher RBC (all *p* < 0.05). Key stereometric parameters used to describe ONH [superior, inferior, nasal, temporal, and mean retinal nerve fiber layer (RNFL) thickness] and macula (macular thickness) increased at high altitude compared with baseline. Most parameters of ONH changed, especially superior, inferior, and mean RNFL thickness (*p* < 0.05). There was a significant correlation between the ratios of RNFL at ONH and HAH [mean thickness (r = 0.246, *p* = 0.01); inferior (r = 0.216, *p* = 0.02); nasal (r = 0.193, *p* = 0.04)]. No associations between parameters of ONH and AMS or LLS were observed.

**Conclusion:** The high-altitude environment can increase RNFL thickness at ONH. Furthermore, we found that the ratios of mean thickness, inferior area, and nasal area correlated positively with HAH, which provides new insights for understanding of the underlying pathological mechanisms of high-altitude retinopathy (HAR).

## Introduction

High-altitude headache (HAH) is the most frequent, most unpleasant, and sometimes only symptom experienced when rapidly ascending from sea level to high altitude (Serrano-Dueñas, 2005; 2007; Burtscher et al., 2011; Carod-Artal, 2014; Wang et al., 2018). According to the widely accepted Lake Louise Consensus scoring system, which was revised in 2018, HAH is also the core symptom of acute mountain sickness (AMS) (Roach et al., 2018). HAH, as defined by the International Headache Society, occurs within 24 h after rapidly ascending to high altitude and is resolved within 8 h after descending; it can be so severe that it can induce life-threatening, high-altitude cerebral edema (HACE) or high-altitude pulmonary edema (HAPE) (Headache Classification Subcommittee of the International Headache Society, 2004; Headache Classification Committee of the International Headache Society, 2013; Lopez et al., 2013; Guo et al., 2017). Given that the incidence of HAH is approximately 80% (Headache Classification Subcommittee of the International Headache Society, 2004; Wilson et al., 2009; Lopez et al., 2013; Marmura and Hernandez, 2015) in subjects who rapidly ascend to high altitude, HAH has become a public health problem that requires urgent resolution (Queiroz and Rapoport, 2007).

The underlying pathological mechanism of HAH is not clear, which is deemed to be an ongoing pathophysiological process of AMS and HACE (Basnyat, 2005). Direct or indirect evidence has shown that increased intracranial pressure (ICP) is the main cause of high-altitude illness, including HAH, AMS, HACE, and high-altitude retinopathy (HAR) (Wilson et al., 2014). Noninvasive methods for direct measuring of ICP do not yet exist. However, parameters of the retina may be ideal candidates for noninvasive indirect assessment of ICP for anatomical (closely adjacent to intracranial tissues) and technical (highly amenable to acquisition) reasons (Clarke et al., 2019). Papilledema (optic disc edema, ODE) refers to the swelling of the optic disc, which clinically serves as a biomarker of increased ICP (Chen and Bhatti, 2019; Eikenberry et al., 2020). ODE was first reported in 1969 as a clinical manifestation of HAR and has been linked with HACE (Singh et al., 1969).

The present study was based on a hypothesis that some retinal parameters may be related to HAH. Accordingly, we explored the association between quantitative measurements of the retina nerve fiber layer (RNFL) and HAH. After collecting demographic information, we performed a repeated measurement of retinal fundus changes and collected physical parameters before and after high-altitude ascent. LLS scores were obtained from 109 healthy young Han Chinese males within 24 h after high-altitude ascent.

## Materials and Methods

### Participants

A total of 109 subjects (all male; mean age: 19.6 years, SD: 1.7 years) lived at 50 m and traveled to Tibet by plane were recruited to our observational cohort study. To participate in the study, subjects had to be healthy Han Chinese men between the age of 18 and 35 years, and no high-altitude exposure in recent 2 years. All of the subjects completed a self-reported questionnaire (structured case report forms, CRFs) to report their disease status and medical history. We did not include participants who had suffered from cardio-cerebrovascular, respiratory, ophthalmic, or migraine diseases, or had had a headache or cold, or were taking any medications during ascent to high altitude. To motivate and recruit subjects, the purpose of the study was explained in detail to all of the subjects who volunteered for participation, and all of the participants signed an informed consent before the examinations. The Human Ethics Committee of Fudan University approved the protocol.

### Study Procedures and Measurements

All of the participants underwent baseline examination 1 week before departure and within 24 h after their arrival at 3,700 m. Demographic (age, body mass index (BMI), smoking and drinking history), physiological [heart rate at rest (HR, beats/min), oxygen saturation (SpO_2_, %)], and hematological data [hemoglobin level (Hb, g/L) and red blood cell count (RBC, *10^9^)] were obtained. To measure the severity of HAH and AMS, the Lake Louise Scoring (LLS) system for acclimatization grading was used. Using the self-reported questionnaire, Lake Louise points were assigned on a 0 to 3 scale for headache, gastrointestinal symptoms, fatigue, and dizziness. All subjects with a headache and LLS ≥3 were considered to have AMS (Roach et al., 2018).

**FIGURE 1:**
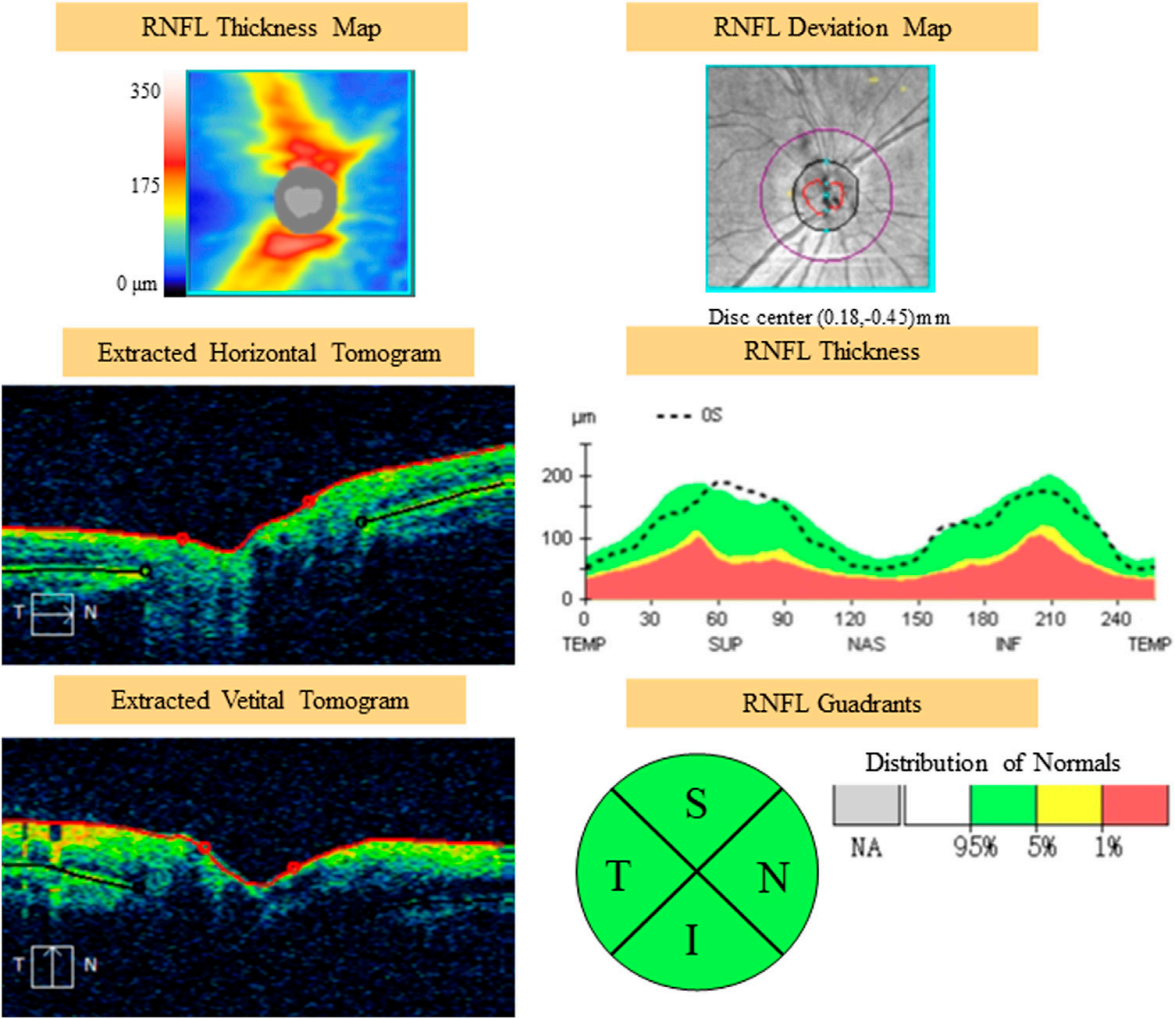
Example of scanning and measurements of RNFL with OCT. RNFL, retinal nerve fiber layer; OCT, optical coherence tomography; S, superior area; I, inferior area; N, nasal area.

**FIGURE 2:**
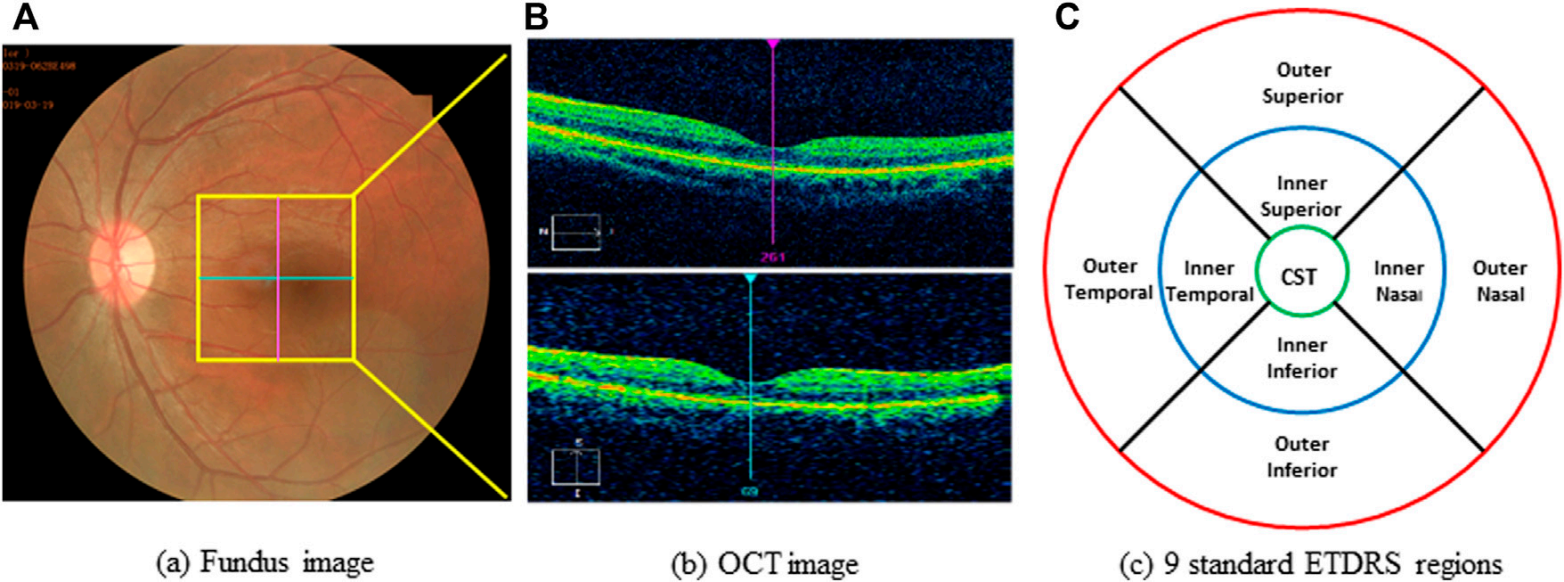
Example of scanning and measurements with OCT. **(A)** funds photograph of subject; yellow box indicates the area of macula. **(B)** OCT image of macula. **(C)** Standard of ETDRS map, divided into 9 regions with 3 concentric rings measuring 1 mm (green ring), 3 mm (blue ring), 6 mm (red ring). ETDRS, early treatment diabetic retinopathy study; OCT, optical coherence tomography; CST, center subfield thickness.

All optical coherence tomography (OCT) scans were acquired by the same operator using the Cirrus OCT device (Cirrus 5,000, Carl Zeiss Meditec Inc. Dublin, California, United States; software version 6.5.0772). The Cirrus HD-OCT 5,000 has an A-scan velocity of 27,000 scans/second with a 5 μm axial resolution and a scanning depth of 2 mm. The instrument uses light of 840 nm wavelength, and images of the optic disc and macula. The optic disc scan was centered on the optic disc (optic disc tube 200 × 200 protocol), while macular scan was centered on the fovea (macular cube 512 × 128 protocol). To eliminate binocular confounding factors, we only included the left eye. Only high-quality images (signal strength ≥6) of the left eye in each participant were taken. The scans were used to measure RNFL thickness and macular thickness. RNFL thickness was automatically calculated by the fast RNFL procedure. The software allows the mapping of thickness data on a quadrant-by-quadrant basis. We considered the average values of three different measurements per quadrant (superior, inferior, nasal, and temporal), and the overall data obtained in all of the quadrants were used to determine the average RNFL thickness (Figure 1). OCT software calculates macular retinal thickness as the distance between the first signal from the inner limiting membrane and the signal from the anterior boundary of the retinal pigment epithelium. The map of the early treatment diabetic retinopathy study (ETDRS) grid is composed of nine sectorial thickness measurements in three concentric circles with diameters of 1 mm, 3 mm, and 6 mm. The area bounded by the outer (6 mm) and middle (3 mm) circles forms the outer ring, and the area bounded by the middle (3 mm) and inner circles (1 mm) forms the inner ring. The central 1-mm circular region represents the foveal area (Figure 2).

### Statistical Analyses

Continuous data were presented as the means ± standard deviations (SDs), and their normality was assessed by the Shapiro–Wilk test. Categorical variables were presented as percentages. Differences between baseline and high altitude were analyzed using a paired-sample Student’s *t* test for Gaussian distribution. When Gaussian distribution was not satisfied for continuous variables, the nonparametric Mann–Whitney *U* test was used to compare the baseline and high-altitude measurements. To evaluate possible correlations between changes at ONH, HAH, or AMS parameters, Spearman correlations were used. The significance level (two-tailed) was set at 0.05.

## Results

### Subjects’ Characteristics

All of the 109 participants completed the self-reported questionnaire, and systemic and hematological measurements at sea level and at high altitude were obtained. The incidence rates of AMS and HAH following acute exposure to high altitude were 44.0 and 67.0%, respectively. AMS scores within 24 h after arriving at high altitude ranged from Lake Louise scores of 0–10, with an average of 2.4. As for the physiological measurements, SpO_2_ decreased from 98.3 to 83.0% (*p* < 0.001), while HR significantly increased from sea level to high altitude. In terms of the hematological measurements, Hb and RBC showed a significant increase (*p* < 0.001; Table 1).

**TABLE 1:** Demographic characteristics and systemic parameters of the study participants (*N* = 109).

Characteristics		Sea Level	High Altitude	*p*
Age, years	—	19.6 (1.7)	—	—
BMI, kg/m^2^	—	23.4 (2.4)	—	—
Smoking, yes (%)	—	70 (64.2%)	—	—
Drinking, yes (%)	—	48 (44.0%)	—	—
LLS	—	0	2.4 (2.0)	—
AMS	—	0	48 (44.0%)	—
Headache	None	0	36 (33.0%)	—
	Mild	0	59 (54.1%)	—
	Moderate	0	11 (10.1%)	—
	Severe	0	3 (2.8%)	—
HR (beats/min)	—	69.0 (8.8)	86.1 (10.5)	<0.001∗∗
SpO_2_ (%)	—	98.3 (1.6)	83.0 (15.1)	<0.001∗∗
Hemoglobin (Hb) [g/L]	—	152.6 (9.4)	159.9 (21.2)	<0.001∗∗
RBC (*10^9^)	—	5.03 (0.28)	5.34 (0.62)	<0.001∗∗

Continuous variables are presented as mean ± standard deviation and categorical variables are presented as percentages, compared using a paired-sample *t* test. Non-normally distributed variables were compared using the Mann–Whitney *U* test.

*p value <0.05; **p value <0.001.

### Changes in RNFL and Macular Thickness in the Peripapillary Sectors and ETDRS Grid, Respectively

**FIGURE 3:**
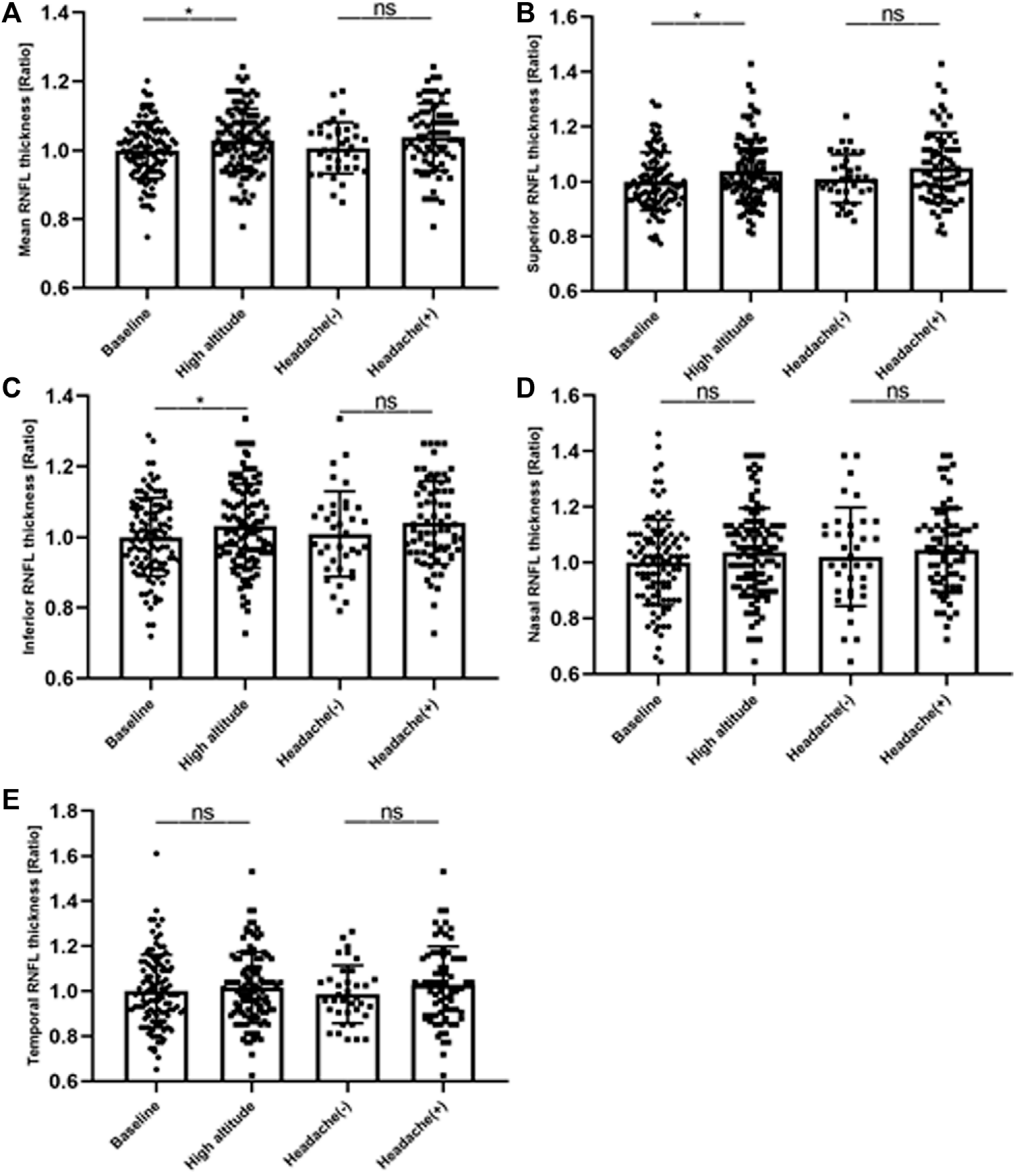
Changes in thickness of RNFL in peripapillary sectors. Intraindividual changes (expressed as ratios) at high altitude. **(A)** mean RFNL thickness, **(B)** superior RNFL thickness **(C)** inferior RNFL thickness, **(D)** nasal RNFL thickness and **(E)** temporal RNFL thickness. RNFL, retinal nerve fiber layer; HAH (+), high altitude headache (HAH); (HAH) (−), none-HAH; *p value indicates p < 0.05; **p < 0.01.

In this study, we investigated the retina of the subjects before and after ascent to high altitude. As shown in Table 2, there were evident changes in the thickness of the RNFL and macular thickness in the participants after their ascent to high altitude. In the optic disc, there was also a significant increase in the thickness of the RNFL in the superior and inferior quadrants and mean RNFL (*p* < 0.05), with an insignificant increase in RNFL thickness in the nasal and temporal quadrants (*p* > 0.05). There was a significant increase in macular thickness in the outer superior and outer nasal quadrants (*p* < 0.05). However, the central subfield thickness exhibited no significant decrease (*p* < 0.05). Changes in thickness of the RNFL in the peripapillary sectors were evident following ascent to high altitude. No significant differences were observed between HAH and non-HAH subjects (Figure 3).

**TABLE 2:** Retinal nerve fiber layer of the optic disc and macular thickness at baseline and after the ascent to high altitude (x ± s, μm).

Characteristics	Parameters	Baseline	High Altitude	*p*
Optic disc	Superior area	131.45 (15.09)	136.34 (16.52)	0.024*
	Inferior area	126.51 (30.65)	131.02 (19.51)	0.028*
	Nasal area	63.83 (10.03)	66.25 (10.41)	0.061
	Temporal area	74.99 (12.14)	76.16 (11.86)	0.437
	Mean thickness	99.35 (8.84)	101.84 (9.08)	0.030*
Macular thickness	Central subfield thickness	243.77 (19.37)	242.95 (19.69)	0.925
	Inner superior	327.32 (30.98)	327.93 (13.16)	0.119
	Inner inferior	319.57 (15.69)	323.78 (13.81)	0.054
	Inner nasal	311.25 (33.24)	312.98 (11.87)	0.106
	Inner temporal	324.76 (14.67)	328.29 (13.48)	0.089
	Outer superior	281.85 (13.46)	287.1 (13.47)	0.005**
	Outer inferior	270.22 (14.14)	273.51 (14.21)	0.089
	Outer nasal	266.14 (12.73)	269.27 (11.83)	0.045*
	Outer temporal	301.84 (18.28)	306.74 (15.09)	0.053
	Mean thickness	99.45 (11.07)	103.26 (8.96)	0.017*

### Relationship Between the Changes of the Optic Disc Parameters and HAH

**FIGURE 4:**
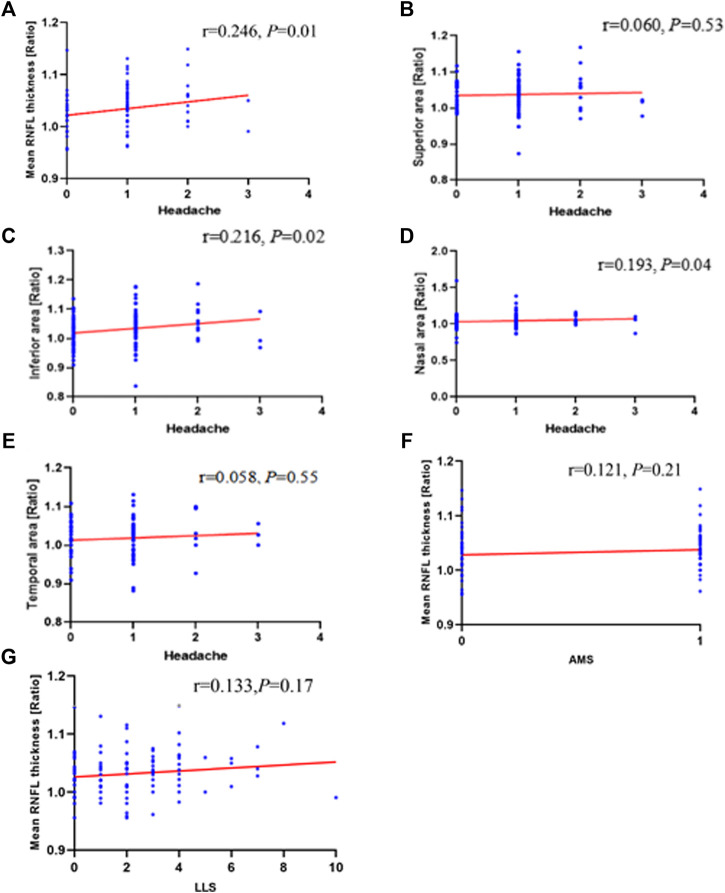
Correlation of changes in ONH with HAH and AMS parameters during high-altitude exposure. Correlation analyses between **(A)** mean retinal nerve fiber layer (RNFL) thickness ratios (*r* = 0 .246; *p* = 0 .01), **(B)** superior area thickness ratios (*r* = 0.060; *p* = 0 .53), **(C)** inferior area thickness ratios (*r* = 0 .216; *p* = 0 .02), **(D)** nasal area thickness ratios (*r* = 0 .193; *p* = 0 .04), **(E)** Temporal area thickness ratios (*r* = 0 .058; *p* = 0 .55) and headache. Mean retinal nerve fiber layer (RNFL) thickness ratios does not correlate with **(F)** AMS (*r* = 0.121; *p* = 0.21) or **(G)** LLS (*r* = 0.133; *p* = 0.17). ONH, optic nerve head; HAH, high-altitude headache; AMS, acute mountain sickness; LLS, Lake Louise score.

Spearman correlation analyses were used to explore the relationships between the optic disc measurements and HAH severity. The correlation analysis revealed a significant correlation between the optic disc measurements and HAH severity. HAH severity significantly correlated with the ratio of mean thickness (r = 0.246, *p* = 0.01 in Figure 4A), inferior thickness (r = 0.216, *p* = 0.02 in Figure 4C), and nasal thickness (r = 0.193, *p* = 0.04 in Figure 4D). The ratios of superior and temporal RNFL thickness did not display a significant correlation with HAH severity (*p* > 0.05 in Figures 4B,E). However, the ratios of mean RNFL thickness did not significantly correlate with AMS (Figure 4F), LLS scores (Figure 4G), gastrointestinal symptoms, fatigue, dizziness, hemoglobin, or RBC (Supplementary Figure S1).

## Discussion

### Alterations in RNFL of the Optic Disc and Macular Parameters

This study was undertaken to objectively quantify structural changes in the optic disc and macula during acute, high-altitude exposure to low atmospheric oxygen, low humidity, and strong ultraviolet radiation. In this study, OCT was used to determine alterations in the retinal morphology, including macular thickness and the RNFL. Evaluating changes in the optic disc that are highly related to the structure of the brain can provide a better understanding of the pathophysiology of HAH.

According to the findings of the RNFL parameters of the optic disc during exposure to high altitude, young Chinese males enrolled in this study had a thicker RNFL in the superior and inferior areas and a thinner RNFL in the nasal and temporal areas, which is consistent with histological changes of the RNFL reported in previous studies (Hsu and Tsai, 2008; Ascaso et al., 2012). A week before the subjects entered Tibet, the RNFL thickness in each quadrant of the optic disc exhibited different degrees of thickening after rapid ascent to high altitude, especially in the superior and inferior quadrants, which is in agreement with Tian et al. (2018). However, we did not assess the effects of long-term exposure to high altitude and subsequent return to low altitude using OCT. In the studies by Tian (Tian et al., 2018) and Willmann (Willmann et al., 2011), the optic nerve did not show permanent damage after short-term exposure, which may indicate that HAR is a benign high-altitude illness.

Our results indicated that the thickness of the macula notably increased, and the outer superior and outer nasal zones were much thicker than at baseline. A study on macular thickness conducted by Fischer et al. quantified macular structure in 14 healthy subjects before and after ascent to high altitude; the authors found a minor increase in total retinal thickness (Fischer et al., 2012). Tian et al. adopted OCT to scan the retinal structure of 91 healthy subjects after 1-month exposure to high altitude (4,600 m above the sea level), and their results indicated a significant increase in RNFL thickness in the superior and inferior zones (Tian et al., 2018). However, previous OCT studies on retinal changes associated with high-altitude exposure and AMS showed no significant alteration in any of the ETDRS subfields (Ascaso et al., 2012; Fischer et al., 2012). Our present findings indicated that acute exposure to high altitude did not result in macular edema, but it did increase the perimacular thickness of ETDRS. This indicates that the macular region has a superior self-regulation potential to meet the demands of oxygenation upon acute exposure to high altitude in healthy subjects.

### The Underlying Mechanism of Increasing RNFL Induced by HAH

**FIGURE 5:**
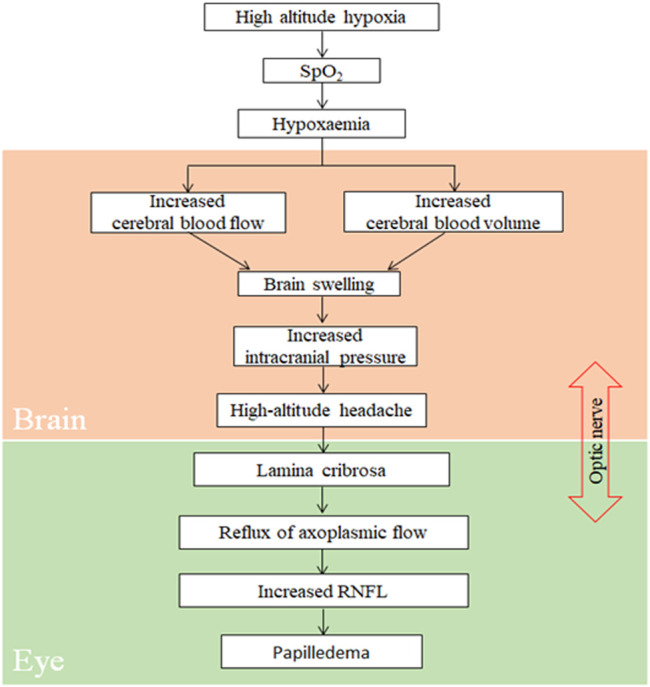
Proposed pathophysiology of retinal nerve fiber layer during exposure to high altitude. Spo2, oxygen saturation; RNFL, retinal nerve fiber layer.

Consistent with the results of other studies (Bian et al., 2013; Shin, 2014), the incidence of HAH, a core symptom of AMS, was 67.0% in our participants. To objectively and quantitatively analyze correlation between parameters of RNFL at ONH and HAH, AMS, or LLS, we monitored changes in the optic disc that were acquired within 24 h of high-altitude exposure, as LLS scores attain their peak values at the same time. According to the proposed model (Figure 5), exposure to high altitude can significantly reduce blood oxygen saturation, thereby inducing hypoxemia (Abdolrahimzadeh et al., 2017). By further stimulating hypoxia through the activation of the corresponding signaling pathway, hypoxemia can result in a significant increase in cerebral blood flow and blood volume, thereby inducing brain swelling and increased ICP (Hackett and Roach, 2001; Rosenblum, 2007; Schoonman et al., 2008; Bailey et al., 2009). The increase in ICP can lead to HAH and elevate the trans-lamina pressure difference (Jonas et al., 2013; Hou et al., 2016). This would directly obstruct axoplasmic flow at ONH (Tso and Hayreh, 1977), and an obstruction to axoplasmic transport could increase RNFL and cause papilledema.

In a large sample, we confirmed that the ratio in the mean nerve fiber layer thickness did not significantly correlate with AMS or LLS scores, which is consistent with the results of previous studies (Willmann et al., 2011; Fischer et al., 2012). Some other studies showed significant positive correlations between AMS and optic disc swelling (Bosch et al., 2008), optic nerve sheath diameter (Bosch et al., 2010), corneal thickness (Bosch et al., 2009), and retinal capillary blood flow (Sutherland et al., 2008). Our study was the first to explore the relationship between changes in RNFL thickness of the peripapillary sectors and HAH; specifically, it illustrated a consistent and robust association between them.

### Limitations

Several limitations to our study should be mentioned. First, the association between alterations of RNFL and HAH was described, but no causal relationship could be determined because of the observational nature of the study. Therefore, longitudinal studies should be conducted to validate our findings in the future. Second, the participants in our study were all young male individuals, which decreases the generalizability of the results. More subjects distributed in different age groups will be recruited in a future study. Third, a longitudinal follow-up study to observe the dynamic changes of the retina at multiple time points and altitudes should be conducted in the future. More parameters of the eyes should be considered, including intraocular pressure (IOP), optic nerve sheath diameter (ONSD), optical coherence tomography angiography (OCTA), and fundus images. Finally, the onset of headache in participants was subjective and not precise. Studies on biomarkers with high sensitivity and specificity for HAH may be desirable in the future investigations.

## Conclusion

In conclusion, our study detected alterations in physiological and retinal parameters after rapid exposure to high altitude (3,700 m above the sea level). We observed a correlation between changes in the RNFL in the optic disc and HAH before and after ascent to 3,700 m, which may offer further insights into the elaborate pathophysiology of papilledema. However, we did not find any correlation between AMS or LLS scores and changes in the optic disc.

## Data Availability

The raw data supporting the conclusion of this article will be made available by the authors, without undue reservation.
