# Supplementary figures and images for: Thickened Retinal Nerve Fiber Layers Associated With High-Altitude Headache
Source: Front Physiol. 2022 May 4;13:864222. doi: 10.3389/fphys.2022.864222 (PMC9114875; doi:10.3389/fphys.2022.864222)

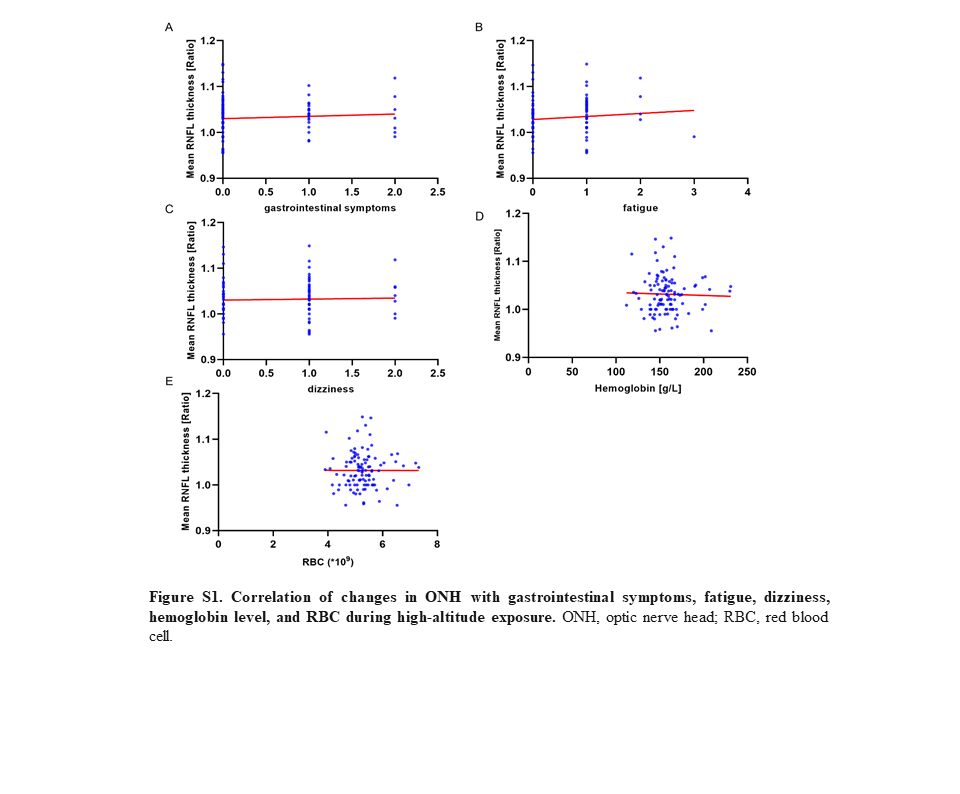

Supplement: Supplementary file 1 [file Image1.TIF]
